# Clinical characteristics of moderate-to-severe thyroid associated ophthalmopathy in 354 Chinese cases

**DOI:** 10.1371/journal.pone.0176064

**Published:** 2017-05-04

**Authors:** Qian Li, Huijing Ye, Yungang Ding, Guo Chen, Zhichang Liu, Jianan Xu, Rongxin Chen, Huasheng Yang

**Affiliations:** 1 Zhongshan Ophthalmic Center, State Key laboratory of Ophthalmology, Sun Yat-sen University, Guangzhou, Guangdong, P.R. China; 2 Qingdao Ludong Eye Hospital, Qingdao, Shandong, P.R. China; Universidade do Porto Faculdade de Medicina, PORTUGAL

## Abstract

Thyroid associated ophthalmopathy (TAO) is an autoimmune inflammatory disorder which disfigures appearance, threatens vision, and results in a pronounced loss of quality of life. The diversity and ethnic difference of the disease manifestations have made it difficult to tailor therapies for each patient. Few studies have analyzed its characteristics in Chinese populations. We therefore enrolled 354 patients with moderate-to-severe TAO from February 2015 to July 2016. A single ophthalmologist consistently performed detailed ophthalmic examinations. Orbital computed tomography or magnetic resonance imaging scans were performed to verify enlarged extraocular muscles. Multiple linear regression was used to analyze the association between sex, age, smoking, family history of thyroid diseases, degree of proptosis and disease severity. The mean age of males (46.56±11.08 years) was significantly higher than that of females (41.39±years), with a female-to-male ratio of 1.09. The females and males between 31~40 and 41~50 years, respectively, had the highest incidence of TAO. 81.48% of the patients suffered hyperthyroidism. TAO was diagnosed either after (47.17%) or simultaneously with thyroid dysfunction (27.68%). Proptosis (91.24%), eyelid retraction (83.33%), together with eyelid swelling (79.38%) and extraocular muscle enlargement (75.42%), were the most common clinical sign. 19.77% of patients manifested lower eyelid retraction. The mean values of exophthalmos and asymmetry on proptosis were 19.94±3.45mm and 2.18±2.06mm, respectively in males, 18.58±3.31mm and 1.61±1.53mm, respectively in females. The severity of disease was significantly associated with male, older age, smoking, family history of thyroid diseases and degree of proptosis. We found several differences in Chinese compared with White. The female-to-male ratio and mean value of exophthalmos were significantly lower than the data of White. Inferior and superior rectus became the most common extraocular muscles. Lower eyelid retraction should be included in diagnostic criteria in Asian patients. Understanding these differences, may allow better identification and treatment for TAO in China.

## Introduction

Thyroid associated ophthalmopathy (TAO) is an autoimmune inflammatory disorder which potentially threatens vision, disfigures appearance and results in a pronounced loss of quality of life. [1, 2] The diversity and ethnic difference of the disease manifestations, combined with the complex pathogenesis, have made it difficult to tailor therapies for each patient, and most of the patients complained about the quality of therapy.[3–6] TAO commonly occurs in patients with Graves’ disease (GD), but patients may also present euthyroid or Hashimotos’ thyroiditis. [7] The exact association between TAO and GD remains elusive. Many investigators believe that loss of immunological tolerance to the thyroid-stimulating hormone receptor (TSHR) underlies the development of disease.[8, 9] Additionally, associations between TSHR antibodies (TRAb), thyroid stimulating immunoglobulin (TSI) and TAO have been reported before. [10, 11]

The clinical course of the disease involves two stages. After a progressive active phase characterized by inflammation and orbital tissue remodeling, the condition gradually stabilizes and eventually trends towards quiescence (inactive phase).[12] Most of the signs and symptoms of TAO, including eyelid retraction, conjunctival congestion, proptosis and restrictive strabismus, appear on binoculus and are closely related to the variation of disease activity. The pivotal pathological characteristics of the disease including inflammatory infiltration, enlargement of soft-tissues and overproduction of the glycosaminoglycan hyaluronan in orbital tissue. [13]

The pathogenesis of the disease arises from a complex interplay of endogenous factors and environmental triggers.[4] Several environmental factors such as sex, age and cigarette smoking are relevant, of which smoking has been clearly illuminated.[14–16] The severity of ophthalmopathy has been found positively associated with advancing age and male sex.[17, 18] Abortive meta-analysis has shown that smoking is an independent risk factor for progression of TAO and GD.[19] And smoking also attenuates effect on the treatment.[20] Therefore, smoking is regarded as the strongest modifiable preventive measurement. Other therapies such as immunosuppression, biological agents, orbital radiotherapy and rehabilitative surgeries have shown promise for treatment.

The epidemiological and clinical features for White patients have been well illustrated, but there is a paucity of literature on TAO in Asian populations, especially Chinese. Previous studies provide limited data on the prevalence, demographics, exophthalmos and ethnic differences in TAO.[7, 21–23] Hence, this study investigates the characteristics of patients with moderate-to-severe TAO, and to give further insight into the clinical-epidemiology of the disease in China.

## Materials and methods

### Patients

All patients diagnosed with moderate-to-severe TAO at Zhongshan Ophthalmic Center from February 2015 to July 2016 were enrolled in this observational study. The study was approved by the Ethics Committee of Zhongshan Ophthalmic Center and all adult patients gave written informed consent to participate. We also obtained written informed consent from the parents of the minors enrolled in our study. The individuals in the study have given written informed consent (as outlined in PLOS consent form) to publish their images. The diagnosis depended on the Bartley criteria.[24] Systematic examinations and orbital computed tomography (CT) or magnetic resonance imaging (MRI) scans were performed to verify the enlarged extraocular muscles and to exclude other confounding causes (e.g., high myopia, orbital tumors, trauma, etc.). All subjects were interviewed by the same ophthalmologist and assisted to complete a structured questionnaire including basic and clinical information.

### Assessment

Recorded demographic and therapeutic information is displayed in Table 1. Patients were stratified into three groups according to their smoking status: never-smokers, former smokers and current smokers with a total consumption of 1–200, 201–400, 401–600, 601–800, and more than 800 cigarettes. We calculated the total consumption of cigarettes as the number of cigarettes smoked per day multiply by the number of years of smoking. The patients who quit smoking for more than 6 months and ≤ 6 months were regarded as former smokers and current smokers, respectively. Features of ophthalmic examination included signs of orbital inflammation, eyelid abnormalities, proptosis and extraocular muscle involvement (Table 2). Lid retraction was classified as upper or lower eyelid retraction only, and retraction of both upper and lower eyelids. Upper eyelid retraction was defined as the eyelid located above the superior corneoscleral limbus in primary position without frontalis muscle contraction. While lower eyelid retraction was noted when the lower eyelid was below the inferior corneoscleral limbus in primary position.[24] Proptosis was measured by Hertel exophthalmometer and recorded as millimeters. The patients were considered as clinically proptosis when exophthalmos was greater than 14mm.

**Table 1:** Demographic characteristics and history of treatments of the patients with moderate to severe TAO.

Characteristics	Value
**Sex (female, %)**	52.26
**Age at diagnosis (years, %)**	
** ≤18**	0.56
** 19–40**	38.42
** 41–60**	52.26
** >60**	8.76
**Smoking status (%)**	
** Never-smokers**	74.01
** Former smokers** ^1^	1.13
** Current smokers** ^2^	
** 1–200 cigarettes**	6.50
** 201–400 cigarettes**	7.91
** 401–600 cigarettes**	5.65
** 601–800 cigarettes**	2.54
** > 800 cigarettes**	2.26
**Family history of autoimmune thyroid diseases (%)**	24.29
**Thyroid function at diagnosis (%)**	
** Hyperthyroidism**	84.18
** Euthyroidism**	11.30
** Hypothyroidism**	4.52
**Thyroid dysfunction duration (months, mean±SD)**	41.93±57.12
**TAO duration (months, mean±SD)**	21.92±32.80
**Bilateral disease (%)**	85.88
**Presenting complaint**	
** Lid swelling**	22.05
** Sclera visible**	4.24
** Prominent eyes**	38.42
** Red eye**	1.69
** Dry eye symptoms**	
** Epiphora**	2.82
** Photophobia**	1.41
** Foreign body sensation**	1.13
** Diplopia**	17.51
** Strabismus**	1.41
** Trichiasis**	0.28
** Blurring of vision**	4.52
** Distending pain of eyes**	4.52
**Antithyroid treatments (%)**	
** Antithyroid drugs**	65.54
** Radioiodine**	16.10
** Thyroidectomy**	5.93
** Untreated**	12.43
**Previous treatments for TAO (%)**	
** Systemic steroids**	38.98
** Orbital steroid injection**	12.81
** Orbital irradiation**	1.41
** Orbital decompression surgery**	13.65
** Untreated**	33.15

TAO: thyroid associated ophthalmopathy.

^1^: The patients who quit smoking for more than 6 months were regard as former smokers.

^2^: The patients who quit smoking for ≤ 6 months were regard as current smokers.

**Table 2:** Clinical features of the patients with moderate to severe TAO.

Ophthalmic characteristics	Male (%)	Female (%)	*P*
**Orbital inflammation signs**			
** Lid swelling**	77.51	81.08	***
** Chemosis**	25.44	15.14	***
** Conjunctival congestion**	42.60	24.86	***
** Caruncular edema**	14.20	9.19	***
** Retrobulbar ache**	8.28	8.11	***
** Oculogyrational ache**	4.73	7.03	***
**Eyelid abnormalities**			
** Lid retraction**			
** Upper eyelid retraction only**	52.66	49.73	0.948
** Lower eyelid retraction only**	24.26	15.68	***
** Upper and lower eyelid retraction**	11.83	12.97	***
** Lid lag**	33.73	24.32	***
** Lagophthalmos**	39.05	34.05	*
**Proptosis**	94.67	88.11	***
**Extraocular muscle involvements**			
** Superior Rectus**	38.92	55.62	0.099
** Inferior Rectus**	54.59	73.37	***
** Medial Rectus**	36.22	47.34	*
** Lateral Rectus**	27.03	20.12	***
** Superior Oblique**	0.00	1.18	**-**
** Levator Palpabrae Muscle**	0.54	1.18	***
** One extraocular muscle**	21.89	11.89	***
** Two extraocular muscles**	31.95	22.70	***
** Three extraocular muscles**	21.30	12.97	***
** Four extraocular muscles**	12.43	16.76	***

***: *P*<0.0001,

*: *P*<0.05.

-: The data was not enough to perform statistical analysis.

**Fig 1:**
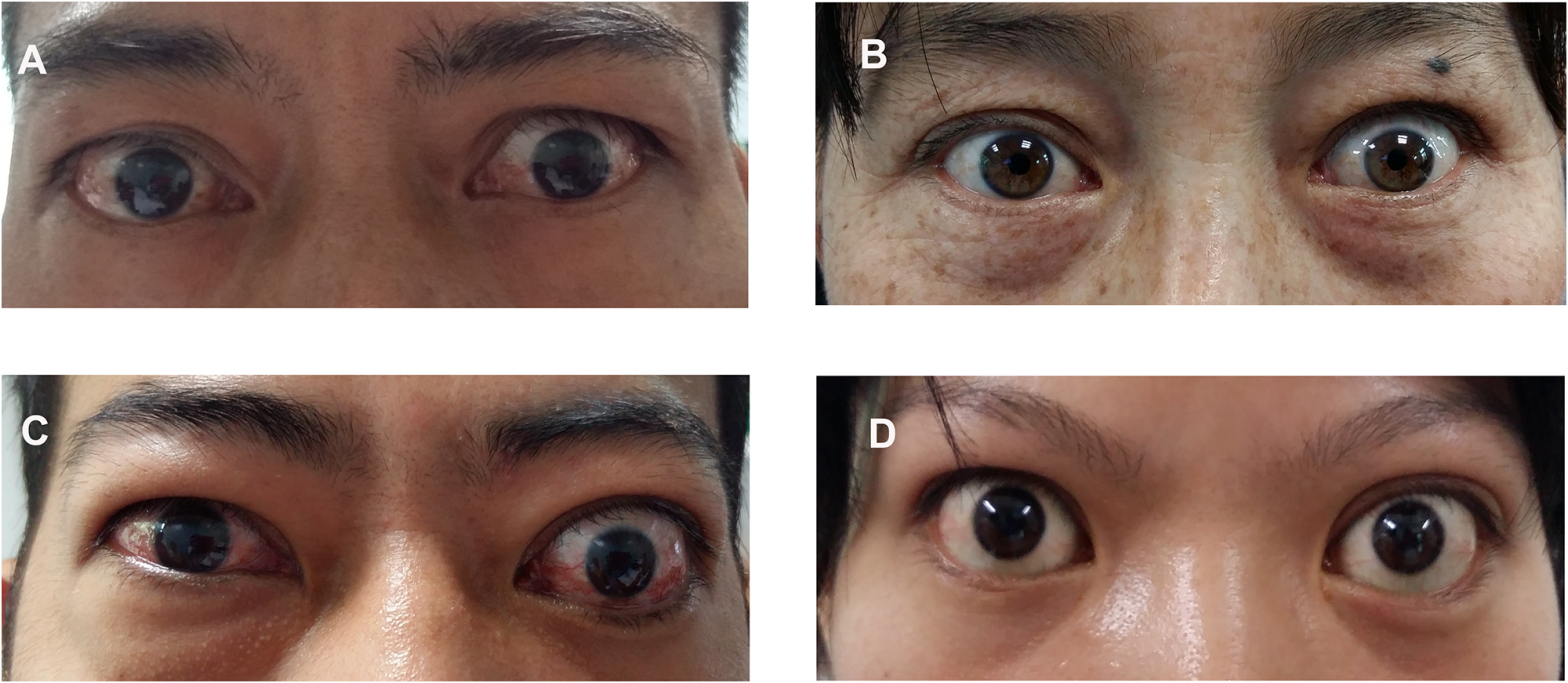
Examples of clinically active and inactive TAO. (A) Moderate active TAO with lid retraction, inflammation of orbital soft tissue for both eyes. (B) Moderate inactive TAO with lid retraction. (C) Severe active TAO with severe binocular soft tissue inflammation, upper eyelid retraction and 25mm of Hertel reading for left eye. (D) Severe inactive TAO with lid retraction and 23mm of exophthalmos.

Patients were defined as active and inactive according to the Clinical Activity Score (CAS) (spontaneous orbital pain, gaze evoked orbital pain, eyelid swelling that is considered to be due to active TAO, eyelid erythema, conjunctival redness that is considered to be due to active TAO, chemosis, inflammation of caruncle, increased of >2mm in proptosis, decrease in uniocular ocular excursion in any one direction of 8°, decrease of acuity equivalent of 1 Snellen line. The last 3 parameters were assessed after 1–3 months follow-up).[25] One point was given for the presence of each parameter mentioned above, the CAS score of >4/10 was defined as active (Fig 1A and 1C), while the score of <4/10 was defined as inactive (Fig 1B and 1D). The disease severity was assessed according to the NOSPECS scheme (mnemonic for no signs or symptoms, only signs, soft tissue involvement, proptosis, extraocular muscle involvement, corneal involvement and sight loss, graded as 0, I, II, III, IV, V, VI).[26] Patients who graded as II-IV and V-VI were classified as moderate and severe, respectively.

### Statistical analyses

Statistical analyses were performed using SPSS version 21.0 (SPSS Inc., Chicago, USA). The *t* test and *χ*^*2*^ test were used to compare clinical features of male and female subjects. The association between sex, age, smoking status, family history of autoimmune thyroid diseases and the degree of proptosis with disease severity was assessed by linear regression analyses. *P*<0.05 indicated statistical significance.

## Results

**Fig 2:**
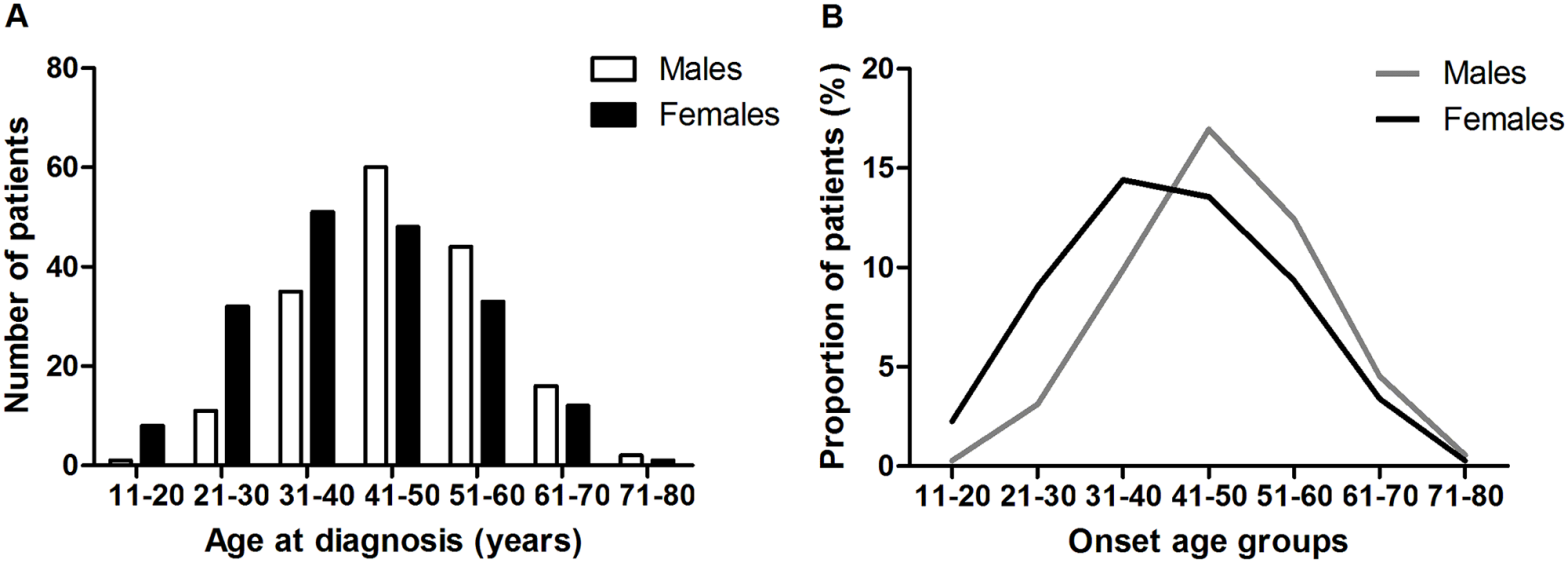
The age at diagnosis of patients with TAO. (A) The bar diagram and (B) linear curves demonstrate the age at diagnosis (years) of patients with TAO.

Three hundred and fifty-four patients with moderate-to-severe TAO were included in the study. The demographic characteristics of the patients were summarized in Table 1. The amount of male and female patients were roughly equal, with a female-to-male ratio of 1.09 (185 were females and 169 were males). The mean (±SD) age of all the patients was 43.86±12.19 years (range, 14–73 years). The mean age of male patients at diagnosis was significantly older than that of females (46.56±11.08 and 41.39±12.63 years for males and females, respectively). The majority of patients were between 41 and 60 years of age (52.26%), followed by 19 and 40 years age group (38.42%). The peak incidence of age was shown in Fig 2(A) and 2(B). The average age of incidence increased and crested at ages 31~40 and 41~50 for females and males, respectively, then decreased progressively.

**Fig 3:**
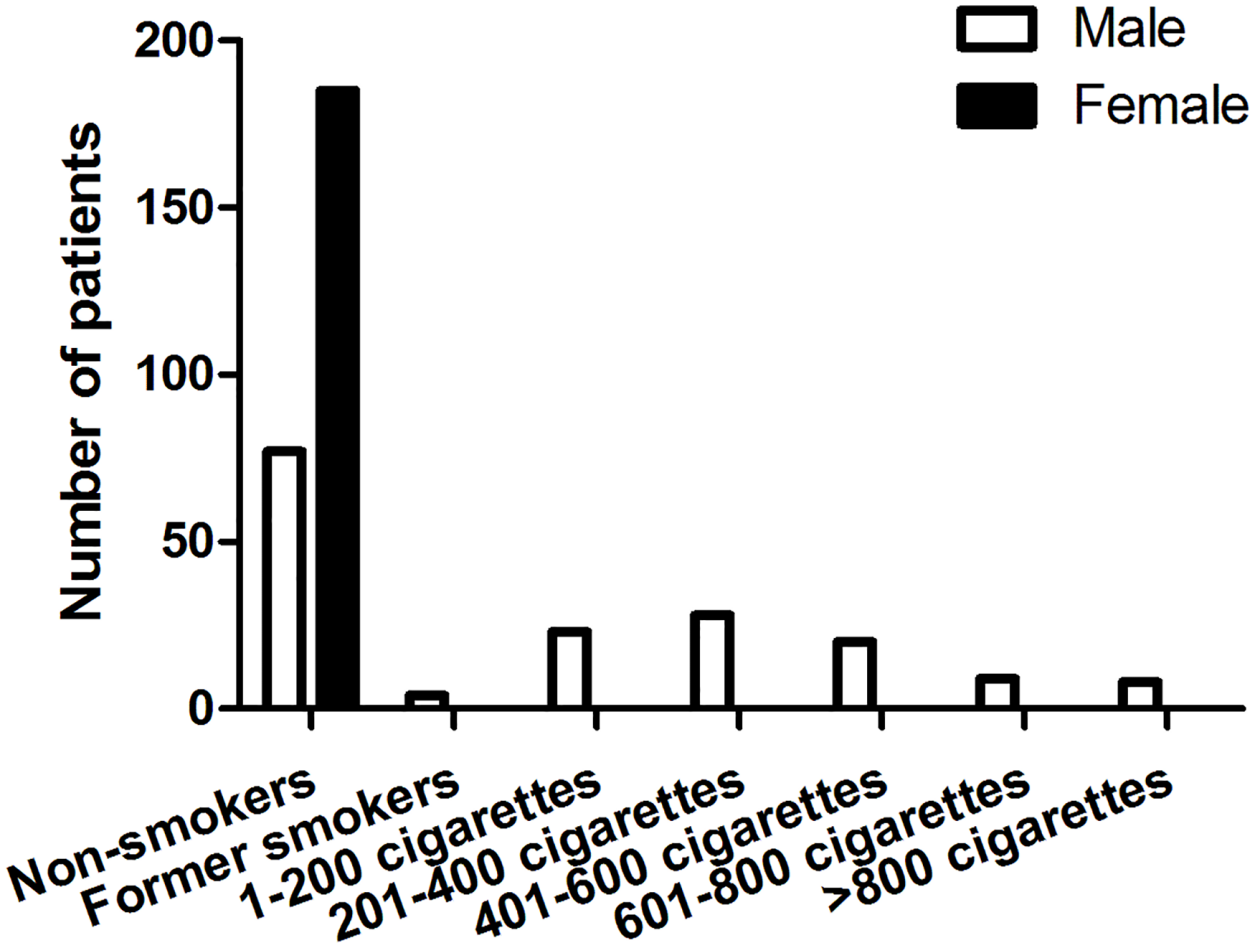
Smoking status of the patients with TAO.

In order to analyze the relative risk for the severity of TAO in relation to cigarettes consumption, patients were divided into three groups. Fig 3 displayed the distribution of patients with moderate-to-severe TAO, based on their smoking status. None of the females were smokers. Among the 169 male patients who were included in the analysis, 52.07% were current smokers. 23 patients consumed 1–200 cigarettes, 28 patients smoked 201–400 cigarettes, 20 patients smoked 401–600 cigarettes, and 9 patients smoked 601–800 cigarettes, 8 patients smoked more than 800 cigarettes until they came to our center. Patients with positive family history of thyroid autoimmune diseases accounted for 24.29%.

**Fig 4:**
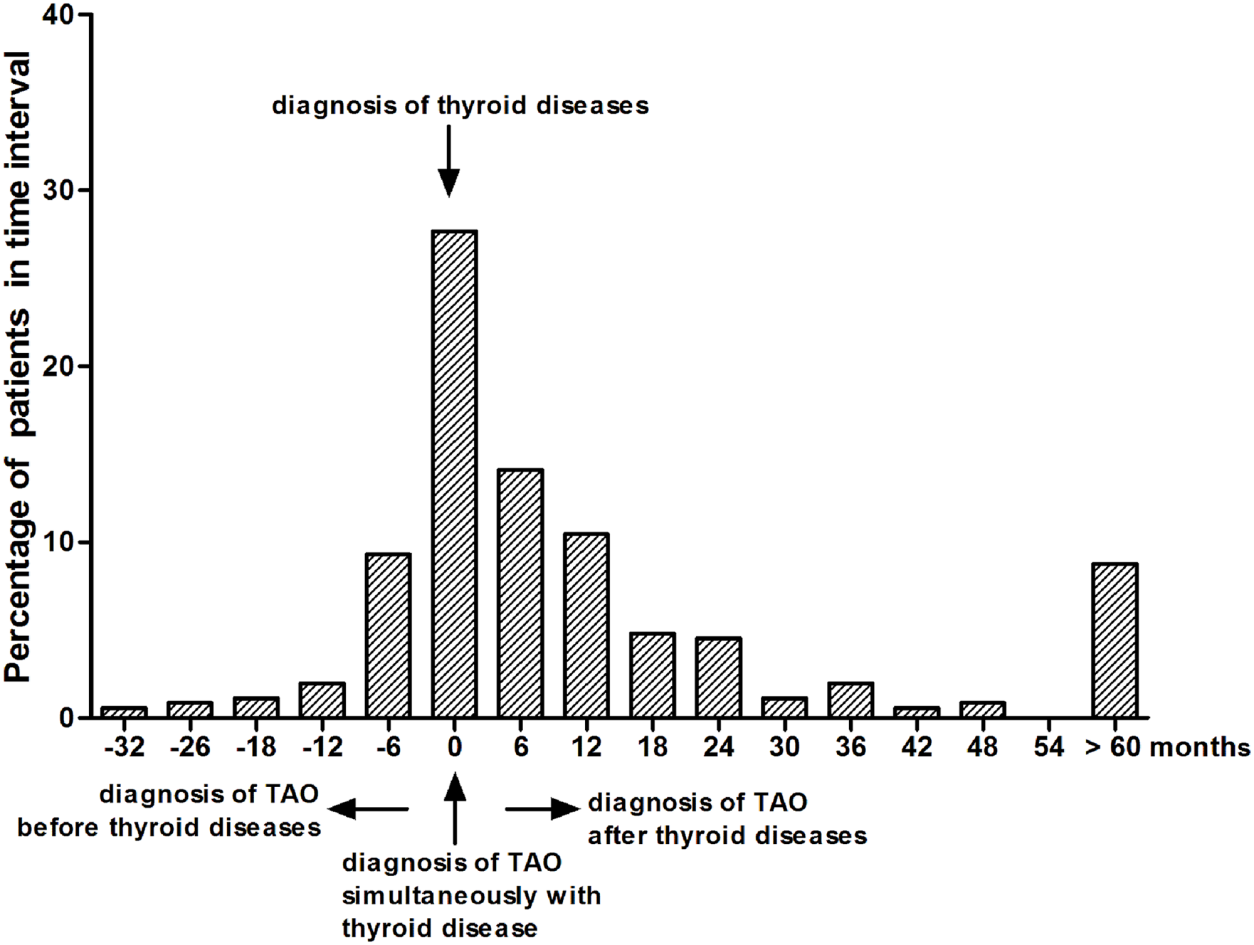
Temporal relationship between the diagnosis of TAO and thyroid dysfunction (hyperthyroidism or hypothyroidism).

Hyperthyroidism was the most common thyroid dysfunction among the patients (84.18%), whereas euthyroidism and hypothyroidism were noted in 40 (11.30%) and 16 (4.52%) patients, respectively. Duration of thyroid dysfunction and ophthalmic symptoms was 41.93±57.12 months (range, 1–360 months) and 21.92±32.80 months (range, 0.2–360 months). Most of the patients were diagnosed within six months and even longer after the beginning of thyroid disease (47.17%), while 27.68% were found to have TAO at the time of occurrence of thyroid autoimmune diseases (Fig 4).

310 (87.57%) patients were receiving antithyroid treatments. The spectrum of management consisted of antithyroid drugs (65.54%), radioiodine (16.10%), and thyroidectomy (5.93%). The ophthalmopathy was treated with systemic steroids (38.98%), orbital steroid injection (12.81%), orbital irradiation (1.41%) and orbital decompression surgery (13.65%).

The majority of patients presented bilateral disease (85.54%). The most common presenting complaint was prominent eyes (38.42%), lid swelling (22.05%) and diplopia (17.51%). In addition, the patients also complained about blurring of vision (4.52%), distending pain of eyes (4.52%) and sclera visible (4.24%). Table 2 exhibited the clinical features of the patients. The presentation of clinical features, except upper eyelid retraction and superior rectus involvement, was significantly different between male and female patients. Most of the patients demonstrated various degree of orbital inflammation signs. More than 80% of patients showed eyelid abnormalities, most frequently, upper eyelid retraction. Lid lag and lagophthalmos were also common among the patients. At diagnosis, 323 patients had propotosis. There was a significant difference of exophthalmometric and asymmetric values between TAO patients and normal subjects (*P*<0.01). The mean Hertel exophalmometric value of normal Chinese adults was 15.75±1.8mm (Table 3). The mean value of absolute difference between bilateral exophalmos was 0.23±0.45mm.[27] 27 The mean value of exophthalmos and the mean value of asymmetry of proptosis for the entire TAO patients was 19.23±3.44 and 1.88±1.83mm. Significant statistically differences were indicated between the mean exophthalmos and the mean value of asymmetry of proptosis in male and female patients (*P*<0.001, *P*<0.01, respectively.). The mean values of exophthalmos were 19.94±3.45mm and 18.58±3.31mm in male and female subjects, respectively. The mean value of asymmetry of proptosis was 2.18±2.06mm in male subjects, and 1.61±1.53mm in female subjects.

**Table 3:** Comparison of exophthalmometric and asymmetric values between normal population and patients with moderate-to-severe TAO.

	Average exophthalmos (mm)	*P*	Average asymmetry (mm)	*P*
**Normal subjects**	15.75±1.8	**	0.23±0.45	**
**TAO patients**	19.23±3.44		1.88±1.83	

**: *P*<0.01.

**Fig 5:**
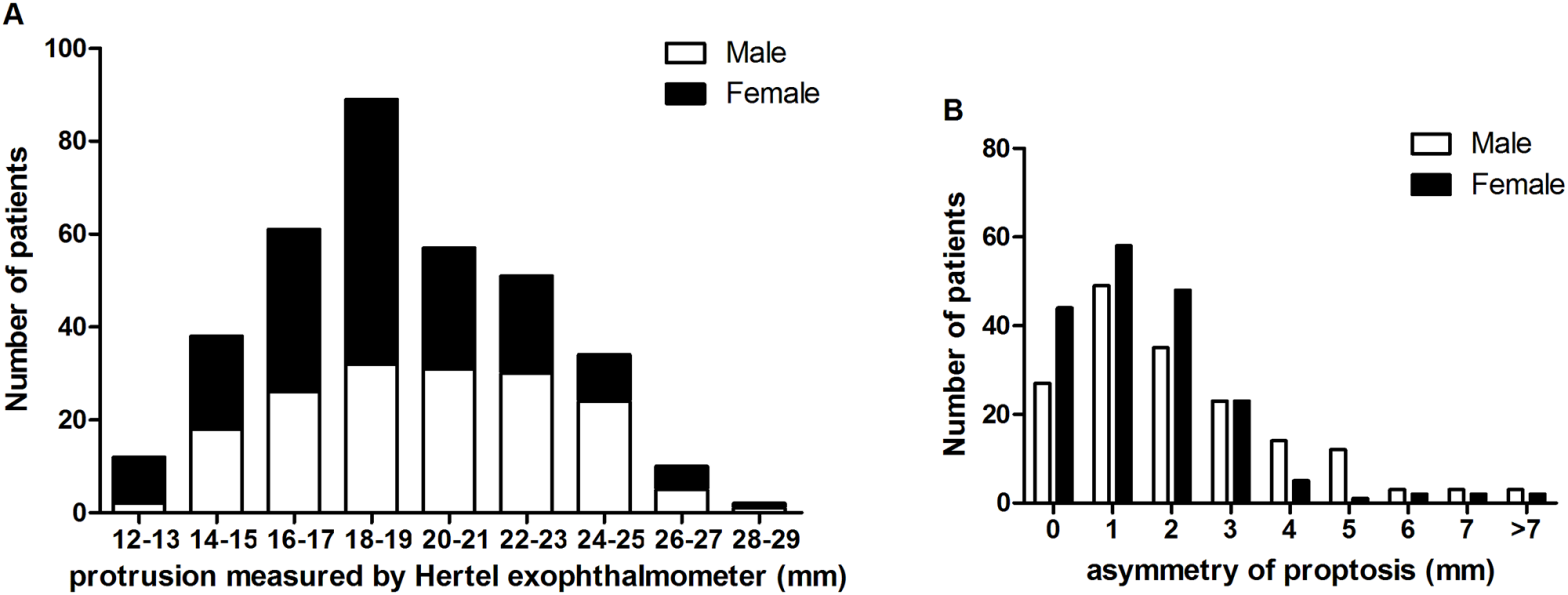
Distribution of exophthalmometric value (worst eye) (A) and asymmetry of proptosis (B) for the patients with moderate-to-severe TAO.

Fig 5 demonstrates the distribution of exophthalmos and comparison of male and female patients with asymmetric proptosis.

**Fig 6:**
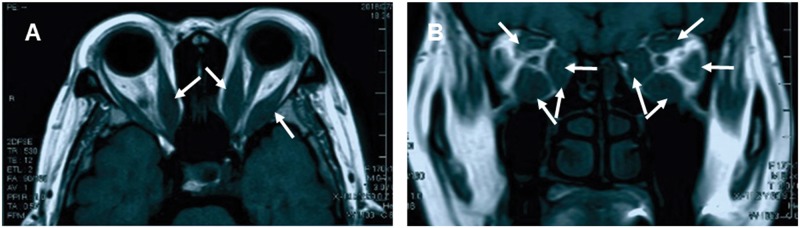
Severe involvement of extraocular muscles on magnetic resonance imaging scan of a patient with TAO. (A) Axial scan demonstrates the fusiform enlargement of extraocular muscles of a patient with thyroid associated ophthalmopathy (white arrows). (B) Coronal scan shows the involvement of muscle belly on magnetic resonance imaging scan of a patient with thyroid associated ophthalmopathy (white arrows).

Definite enlargement of extraocular muscles was noted in 267 patients with TAO in the study. The most frequently involved muscles were the inferior (63.84%) and superior (46.61%) rectus prior to medial (41.53%), lateral rectus (23.73%) and superior oblique (0.56%). In our study sample population, 96 patients had two extraocular muscles enlarged, 60 patients with three extraocular muscles involved and 52 patients with four rectus involvements. Three patients showed levator palpabrae muscle enlargement. Fig 6(A) and 6(B) demonstrated the enlarged extraocular muscles of a patient with moderate TAO.

**Fig 7:**
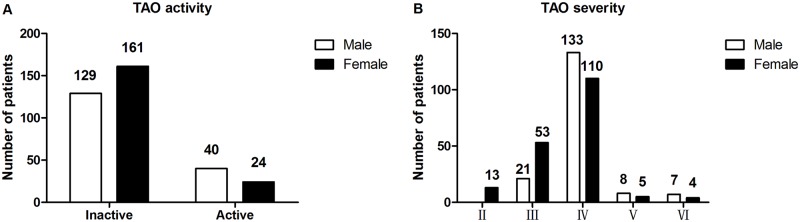
Distribution of disease activity (A) and disease severity (B) in patients with TAO.

The mean CAS and NOSPECS score was 1.95±1.63 (range, 0–8) and 3.82±0.70 (range, 2–6), respectively. 290 (81.92%) patients had inactive disease, and clinically active disease was presented in 64 (18.08%) patients (Table 4). The majority of patients in the study had moderate disease (n = 330), while 24 patients presented severe disease. All of the patients with severe TAO had definite optic neuropathy. Static automated perimetry, VEPs (visual evoked potential) and OCT (optical coherence tomography) were performed in these patients. Visual fields were abnormal in 23 patients, VEP was of abnormal amplitude in 9 and abnormal latency in 21 patients. Comparative thicker retinal nerve fiber layer was found in 8 patients. In addition, 3 patients also presented abnormal color vision. Fig 7 shows the distribution of disease activity and severity in male and female patients. Regression analysis indicated that the severity of disease was significantly associated with the male sex, older age, smoking, family history of autoimmune thyroid diseases and the degree of proptosis (*p*<0.0001, *p*<0.0001, *p* = 0.019, *p* = 0.003, *p*<0.0001, respectively). However, we found the severity of disease was not correlated with total consumption of cigarettes.

**Table 4:** Distribution of disease activity and severity in patients with moderate to severe TAO.

	Percentage (%)		*P*
	Male	Female	
**Disease activity**			
** Active**	23.67	12.97	***
** Inactive**	76.33	87.03	
**Disease severity**			
** II**	0.00	7.03	**-**
** III**	12.43	28.65	***
** IV**	78.70	59.46	***
** V**	4.73	2.70	***
** VI**	4.14	2.16	***

Patients were defined as active and inactive according to the Clinical Activity Score (CAS). One point is given for each parameter, the sum of points defines clinical activity: active if the score is above 4/10 in examination. Disease severity was classified as moderate or severe on the basis of NOSPECS classification: moderate (II-IV: periorbital inflammation presentations proptosis, and eye muscles involvement), and severe (V-VI: corneal involvement or sight loss).

***: *P*<0.0001.

**-**: The data was not enough to perform statistical analysis.

## Discussion

TAO has unique characteristics in different ethnic groups, and its clinical presentations vary by age, gender, smoking status and other external factors.[6] This is the first study that elaborates clinical characteristics of the moderate-to-severe TAO in China. The female-to-male ratio of the patients in the study was 1.09, which was significantly lower than the studies in Sweden (4.2), [28] UK (4.05),[17] European countries (3.40)[7, 29] and Canada (3.45).[18] But the ratio was somewhat close to the study performed in Singapore (1.76)[7] and Malaysia (2.9).[23] We believe the difference came largely from the distinct composition of patients in these studies. In the present study, we included only patients diagnosed with moderate-to-severe TAO, and the quantity of patients with moderate ophthalmopathy had reached 93.22%. In the study of Nigel C et al. 7 and Petros P et al.[29] 29, however, the percentage of mild group in their studies was 71.3% and 60.5%, respectively. Other researchers have focused on the clinical features of Graves’ disease in patients with or without TAO 23 28. A second and also important cause of the low female-to-male ratio arises from the unique health care system and stronger health-conscious of female patients in China. The present health care system in the mainland of China contains primary, second and tertiary referral center. Primary health care provides the first contact, medical examination and diagnosis for people lived in surrounding areas. The second level hospital take charge of further diagnose, treatments and referral. Female patients tend to choose primary and second level hospitals for medical services at the beginning of the illness. Meanwhile, male patients always wait and endure until the disease progresses to an unbearable condition before visiting the doctor. Finally, the male patients in serious condition always concentrated in tertiary hospitals like our center. More importantly, our results also support the view that female-to-male ratio decreases correspondingly with the severity of disease,[3] and the predominance of females over males in the incidence of TAO was considerably less in Asian patients.[23] Besides, we found that the mean age at diagnosis was significantly lower in females than males, which is consistent with the previous studies.[7, 23, 30]

Many studies have confirmed the strong impact of smoking on the incidence, severity, and response to therapies of TAO. Current smokers with GD were more susceptible to develop TAO, and the effect of smoking appeared to be exerting in a dose-dependent manner.[16, 20, 31] Smoking has also been considered increasing the risk for development of severe disease and optic neuropathy.[32] Furthermore, smoking resulted in progression of ophthalmopathy after radioiodine therapy.[15, 33] As expected, we found an association between smoking status and the severity of the disease. In our study, we observed that it was mainly males suffered active and severe TAO (Fig 7A and 7B), largely because they were smokers. Although the mechanism by which smoking affects TAO remains unclear, we emphasized the importance of abstinence from smoking as an extremely effective preventive measure for all TAO patients.

TAO and autoimmune thyroid diseases are closely related diseases. Bartley et al. reported 54% of patients suffered TAO after GD, and only 20.3% experiencing ophthalmic signs and symptoms and thyroid diseases simultaneously.[34] A survey from Bratislava showed that 91% of their patients displayed hyperthyroidism preceding the orbitopathy.[35] In contrast, Wiersinga et al. found that around 40% of patients were diagnosed at the same time of the thyroid dysfunction.[36] In most cases of our study, TAO was diagnosed either after (47.17%) or at the time of the onset of thyroid dysfunction (27.68%). Perros et al. reported that time from first symptom of TAO as perceived by the patient to a diagnosis of TAO first made by a clinician was 9 months.[29] 29 However, in the present study, we found the mean duration of TAO and thyroid dysfunction in Chinese patients was 21.92 and 41.93 months, respectively, which was much longer than European patients. The embarrassing enormous gap reflected failures of cooperation between endocrinologists and ophthalmologists in China. Establishment of a specialized multidisciplinary setting was the key for the patients with TAO to get early diagnosis and treatment. Obviously, we still have a long way to go in this respect.

Upper eyelid retraction and exophthalmos were defined as the two most common signs of TAO in White patients (90% and 62.4%, respectively).[34] However, the prevalence of upper eyelid retraction of Chinese patients was relatively lower (63.56%). The difference may be attributed to several possible reasons. The patients recruited in our study had all been diagnosed as having moderate-to-severe TAO. The anatomically shallower and narrower orbit of Asians compared to White results in a smaller margin reflex distance, hence eyelid retraction may not cause an obvious scleral show.[37] In addition, 19.77% of patients had lower lid retraction in our study which parallels the findings of the Singapore[7] and Malaysia[23] studies. These findings indicated that retraction of lower eyelid is one clinical feature in Asian patients and should be included in the diagnostic criteria. Another common disease manifestation was eyelid swelling that was found in 79.38% of patients in this sample population. Normal orbital volumes vary significantly among races, with Asians having smaller capacity.[38] Therefore any expansion of adipose tissue and enlargement of extraocular muscle will more prominent.

The proptosis caused by enlargement of extraocular muscle and adipose tissue, occurred in 30% to 70% of patients with TAO.[34, 39] Remarkable proptosis may cause secondary exposure keratitis and even corneal ulceration. Patients that develop fibrosis of soft tissues in orbit with reduced exophthalmos may be at great risk of compressive optic neuropathy.[40] Therefore, the change of proptosis may predict the progression of the disease.[41] The ethnical anatomical variability, including the volume or shape of the orbits has been extensively described, and sex difference does exist in some studies.[21, 42], [43] In comparison with previous studies, the mean Hertel reading of our patients was 19.23 mm, which was close to those of Malays (19.4 mm), [23] Singaporean (18.8 mm),[7] Taiwanese (18.32 mm)[22] and Indian (17.7 mm),[44] but lower than those of the White Americans and African Americans.[43] In the present study, we also found a significant sex difference on the exophthalmometer reading. The mean protrusion value of male patients was significantly greater than females. Based on the findings of the present study, the criteria of exophthalmos of White was obviously not appropriate to Chinese population and may result in underdiagnoses. Realizing the ethnic difference of exophthalmometric value would aid in distinguishing the cause of proptosis and treating TAO.

Although clinically unilateral TAO occurs occasionally, the disease is still the primary cause of unilateral exophthalmos in adults.[45] We observed unilateral TAO was found in 14.46% of our patients, which was higher than the previous studies reported a prevalence of 5–10%.[46] Further, our study demonstrates a high mean asymmetry (1.88mm) in moderate-to-severe TAO patients. The average asymmetry of binocular exophthalmos for males was significantly greater than females. A study about exophthalmos of GD patients in Taiwan showed that 6.7% patients presented asymmetric exophthalmos >2mm, compared with 0% in normal subjects.[22] Frueh et al. reported 9% patients with TAO had asymmetric exophthalmos >2mm.[47] We found that 25.71% patients with moderate-to-severe TAO had asymmetric exophthalmos >2mm. It seems that patients with exophthalmos >2mm were more likely attribute to pathological change.

In our study population, we noted 267 patients (75.42%) who by orbital CT or MRI demonstrated different degrees of extraocular muscles enlargement. This result was almost consistent with the previous study conducted in Poland[48] and USA,[49] extraocular muscle involvement was noted in 69.6% and 85% of patients with TAO, respectively. In contrast, a case series study including 10931 Japanese patients had much lower prevalence of extraocular muscle involvement at 40.8%.[50] The difference in composition of study subjects in these two studies may help to explain the discrepancy. Furthermore, we also noted that the most commonly swollen muscle was the inferior rectus. This result was a direct confirmation of other observations made by Sheikh et al., [51] Scott et al., [52] and Ewa et al.[48] However, contrary to the findings of Sheikh et al. and Scott et al., we found the next common involved extraocular muscle was superior rectus, rather than medial rectus. To confirm the reason for these differences would require a larger data set.

It was believed that the severity of TAO has declined in the last decade,[53] and Asian patients appeared to have less severe manifestations of proptosis, periorbital edema and muscle restriction.[21] However, our results did not confirm these findings. Common reasons for this special phenomenon included, first, a lack of awareness of TAO in both patients and doctors, especially those patients living in rural areas and doctors working in primary health care. Thus, the doctors may easily miss the diagnosis of patients in early stages, leading to disease progression. Second, many patients seek medical assistance first in local hospitals. These non-specialized centers tended to leave medical therapies to a tertiary referral center leading to delayed medical treatments and aggravated conditions. As a result, most of the patients with more severe disease were concentrated in tertiary hospitals like our center. Further studies to seek optimal therapeutic schedules based on larger patient numbers are currently being implemented in our center.

## Conclusion

In conclusion, this is the first study which analyzed the clinical features of patients with moderate-to-severe TAO in mainland China. We observed the female-to-male ratio of the patients was significantly lower than the data of White. Inferior and superior rectus became the most frequently involved extraocular muscles. Lower eyelid retraction should be included in the diagnostic criteria in Asian patients with TAO. What’s more, our study demonstrated a relatively lower mean value of exophthalmos in this Chinese sample population. Thus, the criteria of exophthalmos of Chinese still requires further studies. The common distribution of moderate-to-severe TAO in China, as shown by our study, emphasized the importance of interaction between endocrinologists and ophthalmologists. Based on the ethnic variations found in the present study, physicians would be better able to identify the clinical features of TAO and to assess and manage this vexing disease in China.
